# Teleconsultation service to improve healthcare in rural areas: acceptance, organizational impact and appropriateness

**DOI:** 10.1186/1472-6963-9-238

**Published:** 2009-12-18

**Authors:** Paolo Zanaboni, Simonetta Scalvini, Palmira Bernocchi, Gabriella Borghi, Caterina Tridico, Cristina Masella

**Affiliations:** 1 Department of Management, Economics and Industrial Engineering, Politecnico di Milano, Milano, Italy; 2 Salvatore Maugeri Foundation, IRCCS, Telemedicine Service, Lumezzane (Brescia), Italy; 3 Cefriel, Milano, Italy; 4 Lombardy Region - Health Care Directorate, Milano, Italy

## Abstract

**Background:**

Nowadays, new organisational strategies should be indentified to improve primary care and its link with secondary care in terms of efficacy and timeliness of interventions thus preventing unnecessary hospital accesses and costs saving for the health system. The purpose of this study is to assess the effects of the use of teleconsultation by general practitioners in rural areas.

**Methods:**

General practitioners were provided with a teleconsultation service from 2006 to 2008 to obtain a second opinion for cardiac, dermatological and diabetic problems. Access, acceptance, organisational impact, effectiveness and economics data were collected. Clinical and access data were systematically entered in a database while acceptance and organisational data were evaluated through ad hoc questionnaires.

**Results:**

There were 957 teleconsultation contacts which resulted in access to health care services for 812 symptomatic patients living in 30 rural communities. Through the teleconsultation service, 48 general practitioners improved the appropriateness of primary care and the integration with secondary care. In fact, the level of concordance between intentions and consultations for cardiac problems was equal to 9%, in 86% of the cases the service entailed a saving of resources and in 5% of the cases, it improved the timeliness. 95% of the GPs considered the overall quality positively. For a future routine use of this service, trust in specialists, duration and workload of teleconsultations and reimbursement should be taken into account.

**Conclusions:**

Managerial and policy implications emerged mainly related to the support to GPs in the provision of high quality primary care and decision-making processes in promoting similar services.

## Background

National Health Systems (NHSs) are currently facing the challenge of providing better quality health care (HC) and, at the same time, containing costs. Primary care has a relevant role in the current health services scenario. General Practitioners (GPs) - more commonly referred to as Family Medicine in the US, Canada and some European countries [1] - are responsible for the primary evaluation of patients' health status, in particular for the initial steps needed to provide care for any health problem [2] and to supply access to preventive tests and secondary care [3].

The importance of primary care is mainly due to three reasons:

1. GPs contribute to the appropriateness of care, defined as the outcome of a decision-making process that should maximise individual health benefits in view of the available resources [4], with special reference to the growing burden of the elderly and chronically-ill patients [5].

2. Primary care is crucial in the frame of containing costs [6], providing more efficacious actions thus preventing unnecessary hospital accesses, improving timeliness of care and reducing waiting lists [7].

3. GPs have to ensure the best possible access for those patients needing care [2] also providing appropriate care for those who live in remote rural areas and who might have logistic barriers to access secondary care [8], such as distances, transportation costs and a consequent lack of timeliness of care [9]. In fact, accessibility to health care systems (HCS) is a major problem in rural areas [10], with consequent higher disease and mortality rate [11], which, in turn, can also cause rural to urban migration [12].

Nowadays, new organisational strategies should be indentified to improve primary care and its link with secondary care in terms of efficacy and timeliness of interventions [13]. Telemedicine (TM), defined as the use of electronic information communication technologies (ICT) to support HC at distance [14], represents an outstanding solution for improving access in remote areas, currently well accepted by both health professionals [15] and citizens [16]. The most helpful application of TM in primary care is the possibility to provide specialist consultation, usually known as teleconsultation, to the GPs [17,18]. Teleconsultation for GPs has been demonstrated to be feasible [19-21] and effective [22-25], to potentially reduce costs [26-28], to provide organisational benefits [29,30] and to improve patients' satisfaction [31]. However, there are few data about the comprehensive assessment of teleconsultation services that might includes different aspects [32] and provide complete information about their impact.

The aim of the current study is the evaluation of the use of teleconsultation service for GPs in rural areas through a comprehensive assessment which includes: access to HCS, acceptance, organisational impact and utility, effectiveness, and economics of the service.

## Methods

### Description of the TELEMACO Project

The protocol was notified to the Ethics Committee of Local Health Authority of Valle Camonica on 15 May 2007. A previous communication about Teleconsultation Second Opinion was also notified to the Ethics Regional Committee of Lombardy on 8 July 2004 within the SUMMA project [21]. All patients signed an informed consent. The study was conducted in accordance with the principles of the Declaration of Helsinki.

The current study on teleconsultation service for GPs is part of a larger project, the *TELEMACO Project*, combined with three other TM programmes. *TELEMACO Project *[33], promoted by the Lombardy Region - Health Care Directorate, Italy, and funded by the Italian Ministry of Health and the Italian Ministry of Innovation and Technologies, is aimed at supporting small rural communities in mountain valleys and preventing the current rural to urban migration as a result of socioeconomic and infrastructural problems. Another aim of the project is to overcome the difficulties in accessing secondary care in rural areas. In fact, in Italy, admissions to Emergency Departments (EDs) and in-hospital visits are often unnecessary and could be avoided. This would results in waiting lists reduction. Particular attention has been also paid to the promotion of TM networks involving governments, local HC providers, specialised hospitals, small and rural hospitals and GPs in a multidisciplinary and cooperative context. The project has been structured in four different programmes:

1) teleconsultation for GPs;

2) telemonitoring for patients with chronic heart failure (CHF) or chronic obstructive pulmonary disease (COPD) after hospital discharge;

3) teleconsultation on digital images between rural hospitals and specialised hospitals for traumatic brain injury and stroke;

4) cardiology emergency involving the use of TM in ambulances.

Focusing on the teleconsultation for GPs, those working in small communities in the Lombardy Region, Italy, were invited to participate to the *TELEMACO Project *and to use teleconsultations from specialists in cardiology, dermatology, diabetology and pneumology. However, not all the GPs who initially decided to participate in this *TELEMACO Project *actually used the teleconsultation service. Therefore, the GPs were divided into *users *and *non-users*.

The protocol for accessing teleconsultations by GPs was organised as follows. When a patient presented unclear health problems at the GPs' clinic or at home, GPs could ask for a teleconsultation and supply a hospital specialist with the patient's health records and inform the specialist about the observed physical signs and reported symptoms. Teleconsultation then occurred by telephone between GPs and the patient, in the clinic, and the specialist in the hospital. GPs were also invited to use biomedical devices for specific teleconsultations. In particular, for cardiac problems, GPs were equipped with a portable device for ECG recording and remote transmission using an analogic telephone line. For dermatological problems, two digital images (one close to the lesion and another one for overview) had to be performed with a digital camera and were then transmitted by email.

The teleconsultation provision was supported by an external Service Centre (SC) as described elsewhere [19]. Briefly, SC operators receive the GP's request, store the transmitted data on a dedicated database, connect the GP to a specialist on call and record the results of the teleconsultation.

### Assessment Model

Although TM has several applications, it is a debated issue by the scientific community [34] since its benefits are still to be demonstrated [35]. Besides the emphasis on a holistic view of TM evaluation [36], in addition to the need to prove its effectiveness, several strict frameworks have been proposed for the evaluation of TM applications [37-39]. According to these models, a comprehensive evaluation of the TM service (TMS) has been conducted through a multidimensional assessment dealing with the following dimensions:

*(i) Access*. The main dimension used to measure access in TM is its actual utilisation, as suggested by the Institute of Medicine (IOM) [40] and confirmed by other reviews [41]. Therefore, access has been firstly measured through the number of GPs and patients who effectively used and benefited from TMS in the districts involved in the project. Additionally, in HC, access can include a set of specific dimensions (availability, accessibility, accommodation, affordability and acceptability) describing the fit between the patient and the system [42,43]. In particular, geographic accessibility has been measured through the number of inhabitants of the small communities where GPs receive their patients. Additionally, distances and travel times between those communities and the nearest HC provider where patients could receive visits or examinations, hospitalisations or admissions to EDs have been computed. The hypothesis of this optimistic scenario is that patients would actually access the nearest structures.

*(ii) Acceptance *has been evaluated in details by administering exclusively the *users *GP a questionnaire at the end of the project. The questionnaire was structured in 10 questions which examined the overall quality of the service, the contact with SC, the clinical website, the quality of consultations, the accuracy of suggestions, the equipment for data transmission, the duration of teleconsultations, the adherence to suggestions, the impact on solving clinical problems and the training utility. Each question had a 5-point Likert scale, where 1 and 5 represent the lowest and the highest levels of satisfaction, respectively.

*(iii) Organisational impact *has been evaluated by another questionnaire administered to both *users *and *non-users *GPs at the end of the project. The first question was aimed at comprehending the perceived usefulness of TMS in relation to the three specialties under consideration (*useful *or *useless*). In order to evaluate possible difference among specialties, GPs were also asked to explain, through open questions, why teleconsultations were considered either *useful *or *useless*. GPs were also asked to score three main benefits of TMS (timeliness of care, patient's transportation saving, resource saving) and four items related to possible improvement (relationship and trust in the specialist, duration of teleconsultation, teleconsultation support staff, reimbursement for GPs), considered necessary for a larger diffusion of the TMS through a 5-point Likert scale, where 1 and 5 represent the lowest and the highest scores, respectively.

*(iv) Effectiveness*. In the current study, effectiveness has been evaluated in terms of appropriateness of the service for the management of patients, through the correspondence [24] between the action the GP would have chosen without the teleconsultation and the actual decision taken after specialist's suggestions [44]. The protocol anticipated that after the conclusion of each teleconsultation, GPs were asked to answer the two following questions: (i) *Which action would you have taken without the TMS? *(ii) *Which action have you taken after receiving the teleconsultation? *Since the GP is responsible for prescribing hospital visits and diagnostic examinations, data related to preventive tests and specialist visits are accurate. Conversely, admissions to EDs and hospitalisations have not been validated through a follow-up.

*(v) Economics *considered both the HCS and the patient's perspectives. From the HCS's perspective, the analysis has been specifically focused on direct costs and savings related to in-hospital visits, diagnostic examinations and teleconsultations. The cost for teleconsultations is €18.16 and includes all the direct costs of the service. The economic value of a visit is €34.12 and includes the cost for a first visit and the cost for the ECG. Costs of diagnostic examinations were selected according to the type of requests. Economic analysis on admissions to EDs and hospitalisations was not possible since detailed information was not systematically collected through a follow-up.

From the patient's perspective, saving has been related to the direct costs of travels. Indirect costs have not been collected.

## Results

### Use of Telemedicine Service

Table 1 shows the types of teleconsultations conducted in small rural communities in mountain valleys of Lombardy between March 2006 and April 2008. There were 957 teleconsultations on a total of 812 patients: 927 of these were requested by GPs for cardiac problems, 18 and 12 for dermatology and diabetology specialties, respectively. Teleconsultations for pulmonary problems were not used by any GP.

**Table 1:** Characteristics of the Teleconsultations

*Specialty*	*Number of TC*	*Duration of TC*
*Cardiac*	927	5.4 ± 3.7 minutes
*Dermatological*	18	9.5 ± 3.7 minutes
*Diabetic*	12	9.2 ± 4.4 minutes

**Total TC**	**957**	**5.4 ± 3.7 minutes**

TC indicates Teleconsultations

The mean duration of a teleconsultation differed according to the type of clinical problem: cardiac teleconsultation was the shortest (mean 5.4 ± 3.7 min) while dermatological and diabetic teleconsultations required 9.5 ± 3.7 and 9.2 ± 4.4 min, respectively.

### Access

*TELEMACO Project *initially involved 94 GPs who were interested in the use of TMS. At the end of the 2-year implementation period, 48 GPs who practice in small mountain communities in the Lombardy Region, accessed teleconsultations with a use rate of 52%. The mean number of teleconsultations per *user *GP was 19.9. As published in a previous paper [21], tendency to form associations and attitude to utilise ICT were reported by GPs who used the service. Conversely, no statistically significant difference between *users *and *non-users *GPs was evidenced in terms of number of patients, post-graduate study and areas. In addition, 24 out of the 48 GPs who used TMS had already acquired knowledge on the system by participating to previous projects: this experience probably led to a better and larger use of TMS.

As far as the patients are concerned, 812 patients (407 male and 405 female) who presented cardiac, dermatological and diabetic problems benefited from TMS. The mean age of the population was 61.6 ± 19.4 years, significantly higher in females (66.9 ± 18.0 years) if compared with males (56.4 ± 19.4 years). Due to the possibility of using the service more than once during the study period, each patient made on average of 1.6 teleconsultations with the GP's clinic and this highlights the need for continuity of care in chronically ill patients.

Finally, teleconsultations took place in 30 small rural communities, with an average population of 3,723 inhabitants. The average distance between the communities and the nearest HC provider, either a local outpatient clinic or a hospital, where patients could receive visits and examinations, was 7.5 ± 6.2 km, corresponding to 12.2 ± 8.2 min of one-way travel. Additionally, the average distance between the communities and the nearest hospital with an ED was 12.5 ± 9.3 km, corresponding to 17.6 ± 10.2 min of one-way travel. Since this optimistic scenario is based on the hypothesis of access to the nearest clinics and does not take into account common unexpected events (e.g. traffic), real distances and travel times are underestimated.

### Acceptance

41 (85%) out of the 48 *users *GPs completed the acceptance questionnaire administered at the end of the project (Table 2). Most of the GPs were very satisfied by the specialist consultations (Q1), and this, in turn, reflects a high perception of the quality offered by TMS (far beyond their initial expectations) without any negative answer. The connection with SC (Q2) was completely satisfactory for all the GPs justifying future involvement in the frame of teleconsultations. SC also offered a clinical website where data about teleconsultations were collected and which was well accepted by *users *GPs (Q3). SC also provided the equipment necessary for remote data transmission for the different specialties and satisfaction about this item resulted to be slightly higher (Q4). The quality of specialists' suggestions about clinical problems was considered good (Q5). However, some GPs did not consider the service essential, due to the traditional practice of directly prescribing an in-clinic visit or a hospital admission. In addition, the clarity of suggestions, a key element for the correct functioning of the service, resulted to be satisfactory or, more frequently, even very satisfactory (Q6), reflecting the GPs' high level of trust in the specialists. Moreover, the perception concerning the duration of teleconsultations reflects that the time required for TMS use was always adequate (Q7) in respect to the duration of a traditional in-clinic visit and the long waiting lists. Finally, the last three items synthesise the impact of teleconsultations on the GPs' activity. The agreement between specialists and GPs (Q8) was very high, and there were no cases in which GPs took any action different from the suggestion of the specialist, thus reflecting the remarkable decisional utility of the TMS. The teleconsultations effectively succeeded in solving most of patients' clinical problems (Q9), with a consequent high level of clinical utility and effectiveness of the service. A similar level of satisfaction was observed for the training utility (Q10) of the GPs, with a consequent improvement in knowledge concerning cardiology, dermatology and diabetology topics with a positive impact for future management of similar problems.

**Table 2:** Acceptance of the Teleconsultations by GPs

*Questions* *(scores)*	*1*	*2*	*3*	*4*	*5*	*Total*
Contact with SC	0.0%	0.0%	0.0%	17.1%	82.9%	4.8
Clinical Website	4.8%	28.6%	19.0%	38.1%	9.5%	3.2
Equipment for Data Transmission	0.0%	0.0%	4.9%	65.8%	29.3%	3.7
Quality of Consultations	0.0%	5.0%	27.5%	60.0%	7.5%	4.7
Clarity of Suggestions	0.0%	2.4%	0.0%	22.0%	76.6%	4.2
Duration of TC	0.0%	0.0%	80.5%	12.2%	7.3%	3.3
Adherence to Suggestions	0.0%	0.0%	9.8%	39.0%	51.2%	4.4
Impact on Solving Problems	0.0%	2.4%	12.2%	63.4%	22.0%	4.0
Training Utility	0.0%	2.5%	15.0%	65.0%	17.5%	4.0

**Overall Quality**	**0.0%**	**0.0%**	**4.9%**	**56.1%**	**39.0%**	**4.3**

Score: 1 = low; 5 = high; SC indicates Service Centre; TC, Teleconsultations

### Organisational Impact

94 GPs initially decided to participate. At the end of the project, all of them were asked to fill-in the questionnaire which was designed to obtain detailed information on organisational issues concerning the teleconsultation service, regardless of whether they belonged to the *user *or *non-user *categories. 60 GPs (64%) completed the questionnaire. As shown in Table 3, cardiology is perceived to be much more useful for clinical practice than dermatology or diabetology. GPs considered all specialties of useful benefits (ease of use, timeliness and accuracy of the service, reduction of waiting lists, transportation and costs). However, dermatological and diabetic teleconsultations had more barriers due to the preference of a face-to-face visit at the specialist's clinic.

**Table 3:** Perceived Utility of the Teleconsultations

	*Perceived Utility*		
***Specialty***	***Usefulness***	***Reasons for Usefulness***	***Reasons for Uselessness***

***Cardiology***	**95%**	reduction of waiting lists, ease use, timeliness of TC, management of emergencies and unnecessary actions, specialists' skills and expertise	problems for acute events that require immediate hospitalisation

***Dermatology***	**64%**	transportation, waiting lists, avoid unnecessary visits, functional and accurate consultations even though more complex than cardiac TC	too much time for technical-operative problems, difficult clinical evaluation, face-to-face visits often preferred

***Diabetology***	**71%**	handiness and timeliness of care especially for infrequent emergencies and complicated situations, optimisation of therapy	low tendency of requesting diabetic consultations, face-to-face visits often preferred

TC indicates Teleconsultations

Table 4 shows the organisational benefits which were higher for cardiology than for the other specialties, which, anyhow, registered satisfactory results. The main benefit was a cut in patients' transportation, with a consequent improvement in quality of life and cost-savings, even from the caregiver's perspective. Furthermore, GPs succeeded in improving the patient's timeliness of care through the provision of teleconsultations and, at the same time, in reducing the need for secondary care, hospitalisations and admission to EDs. Finally, all the four items related to possible improvement in supporting the diffusion of the TMS have been perceived as important for the future application of the teleconsultation service, with the first item receiving the highest score. Relationship and trust in the specialists represent key elements directly depending on well-known skills and expertise. Time is another remarkable issue: besides the fast cardiac teleconsultations, the duration of the other specialist consultations could be improved in order to limit the burden of the GP's activities. As an alternative, the introduction of additional staff, such as nurses or assistants, could improve the service. Finally, a reimbursement for the activity conducted by GPs could contribute to improve the teleconsultations use, in order to involve also *non-user *GPs, and to increase the number of remotely managed problems. In fact, a reimbursement was anticipated only for the specialists who provided teleconsultation.

**Table 4:** Organisational Benefits, Barriers and Control Improvement by Teleconsultations

*Items*		*Score*
***Organisational Benefits (Cardiology)***		
Timeliness of Care	(1-5)	4.3
Patient's Transportation Saving	(1-5)	4.5
Resource Saving	(1-5)	4.1
***Organisational Benefits (Dermatology)***		
Timeliness of Care	(1-5)	3.2
Patient's Transportation Saving	(1-5)	3.5
Resource Saving	(1-5)	3.5
***Organisational Benefits (Diabetology)***		
Timeliness of Care	(1-5)	3.4
Patient's Transportation Saving	(1-5)	3.3
Resource Saving	(1-5)	3.3
***Barriers and Control Improvement***		
Contact of and Trust in the Specialist	(1-5)	3.7
Duration of TC	(1-5)	3.6
Support Staff for TC	(1-5)	3.4
Reimbursement for GP	(1-5)	3.5

TC indicates Teleconsultations; GP, General Practitioner

### Effectiveness

Table 5 details the type of the 927 cardiac teleconsultations. In the majority of the teleconsultations (844 - 91%), the specialist modified the GP's decision. A saving of NHS resources and a consequent improvement in efficiency could be recorded in 797 cases (86%) while in 47 cases (5%) there was an improvement in timeliness. In the most common situation (613 out of the 927 requests), GPs usually would have prescribed a visit to the patient. However, after teleconsultation, only 7 appointments were actually required in order to better evaluate the patients' conditions through a face-to-face visit. The majority of these specialist consultations implied a lower need of hospital resources. In 397 cases, no actions were indicated, with a consequent relevant cost saving. On the contrary, six patients effectively needed hospitalisation and 22 patients were in such critical conditions requiring urgent admission to EDs.

**Table 5:** Details on Cardiac Teleconsultations

	*Specialist Consultation*							
	
*GP's Decision*	*ED*	*Hosp*	*In-Clinic*	*Diagnostic*	*None*	*Therapy*	*Contact*	*TOTAL*
ED	38 (20.0%)	4 (2.1%)	6 (3.2%)	36 (18.9%)	59 (31.3%)	35 (18.4%)	12 (6.3%)	190
Hosp	2 (33.3%)	0 (0%)	0 (0%)	1 (16.7%)	2 (33.3%)	1 (16.7%)	0 (0%)	6
In-clinic	22 (3.6%)	6 (1.0%)	7 (1.1%)	65 (10.6%)	397 64.8%)	71 (11.6%)	45 (7.3%)	613
Diagnostic	5 (7.8%)	0 (0%)	0 (0%)	9 (14.1%)	37 (57.8%)	7 (10.9%)	6 (9.4%)	64
None	1 (1.9%)	0 (0%)	0 (0%)	11 (20.4%)	29 (53.7%)	9 (16.7%)	4 (7.4%)	54

**TOTAL**	**68**	**10**	**13**	**122**	**524**	**123**	**67**	**927**

GP indicates General Practitioner; ED, Emergency Departments; Hosp, Hospitalisations; In-Clinic, In-Clinic Visits; Diagnostic, Need for Diagnostics; None, No Action; Therapy, Therapy Change; Contact, Contact Reprogramming

### Economics

Due to the majority of TMS use in cardiology, the analysis on the economics dimension is specifically related to this field. From the HCS's perspective, Table 2 shows how the 927 cardiac teleconsultations allowed avoiding 600 cardiac visits (only 13 visits were actually performed out of the 613 requested by GPs) and 122 admissions to EDs. Conversely, the teleconsultation resulted in additional 58 diagnostic examinations and six hospitalisations. The economic analysis specifically focuses on direct costs and savings related to in-clinic visits, diagnostic examinations and teleconsultations. From the one hand, the amount of costs is €16,834.32 for the use of teleconsultations and €5,445.81 for the additional diagnostic examinations. From the other hand, the amount of direct savings for in-clinic visits is €20,472.00. Therefore, the cost for 927 cardiac teleconsultations was €1,808.13 in addition to the traditional practice performed without the TMS, resulting in a considerable economic balance which should not anyway be considered the main objective of the whole project: once the rationalisation of resources is reached, more remarkable benefits will come from the improvement of the appropriateness of patients' care and the management of clinical problems.

From the patient perspective, the 927 teleconsultations requested for 812 patients at the GPs' clinic in the rural communities resulted in a direct savings equal to €3,700.56. In particular, €1,000.06 and €2,700.50 were saved for avoided travelling for admissions to EDs and hospitalisations and for in-clinic visits and diagnostic examinations, respectively.

## Discussion

The current study was performed to assess the process of TM-provided teleconsultation service for GPs.

The improvement of the appropriateness of care - as also underlined by the GPs' acceptance with a clear integration between primary and secondary care [45] - constitutes the managerial and practical relevance of TMS. In fact, the use of teleconsultations succeeded in modifying the behaviour of the GPs [46], who are often reluctant to adopt ICT in their practice [47,48], leading to different types of collaborative work [49] with a consequent improvement in the quality of care with respect to the GP's responsibility.

Teleconsultation has proved to be effective in cardiology, with a remarkable increase in the appropriateness of primary care and integration with secondary care, in keeping with previous studies [22,23,25]. The level of concordance between intentions and consultations for cardiac problems was equal to 9%, in 86% of the cases the service entailed a saving of resources, and in 5% of the cases the timeliness of care improved. The economic analysis, which included direct costs and savings related to in-clinic visits, diagnostic examinations and cardiac teleconsultations, showed a substantial economic balance. However, since savings related to admissions to EDs and hospitalisations have not been included, the real economic benefits can be considered to be higher.

The relevance of TM application is mainly linked to its possible widespread use that, in our study, improved the access to HC for 812 patients living in 30 small rural communities [9]. However, Italy is a country where a teleconsultation service is mainly supplementary rather than specifically alternative. Benefits deriving from geographical access could be higher in countries characterised by a greater *physical *distance between primary and secondary care.

Some barriers also derive from the actual organisational of the general practice. For future use of the service in routine clinical practice in regional and NHS care settings, issues concerning trust in the specialist, duration and workload of teleconsultations [29] have to be taken into account.

This study has also relevant social and politic implications. The positive results reached after a 2-year implementation period confirm the feasibility of new HCS which support primary care [19] and improve equal access to HCS [10]. These services are meant as TM-based networks which involve governments, HC providers, GPs and also private SCs in a multidisciplinary and cooperative context. Since this study was conducted in small rural communities in Italy, the study design and consequent results could firstly support decision makers in similar countries or geographic settings where a teleconsultation service is mainly supplementary rather than specifically alternative. Additionally, benefits to geographical access could be higher in countries characterised by a greater distance between primary and secondary care in order to support the problems which affect rural areas [8], to contrast rural to urban migration [12] or more simply to acquire evidence-based knowledge.

The main limitation of this study is the focus on the teleconsultations rather than on the patients' continuity of care concerning the collection of health-related data. In fact, patients' follow-up could help the comprehension of the effectiveness of the service not only in terms of a better efficiency but also in terms of clinical outcomes. Another limitation is related to the economics dimension. In fact, costs and savings related to admissions to EDs and hospitalisations were not included since detailed information was not systematically collected through a follow-up.

## Conclusions

The current study has shown how a teleconsultation service through TM can help GPs in the management of their patients and provide high quality primary care in order to specifically solve cardiac problems. Further research should be conducted on the impact of teleconsultations as a common health service: the evidences shown in an experimental setting should be evaluated on a daily basis in order to define the possible daily application of the service.

## Competing interests

The authors declare that they have no competing interests.

## Authors' contributions

CM and PZ performed data analysis and assessment of the teleconsultation service. SS and PB contributed to the collection and elaboration of data and to the literature review. CT and GB contributed to define the objectives of the manuscript and authorised data divulgation. All the authors revised and approved the last version of the manuscript.

## Pre-publication history

The pre-publication history for this paper can be accessed here:

http://www.biomedcentral.com/1472-6963/9/238/prepub
